# Radical scavenging activity of plant extracts from improved processing

**DOI:** 10.1016/j.heliyon.2019.e02763

**Published:** 2019-11-14

**Authors:** Adél Szerlauth, Szabolcs Muráth, Sándor Viski, Istvan Szilagyi

**Affiliations:** a MTA-SZTE Lendület Biocolloids Research Group, Department of Physical Chemistry and Materials Science, University of Szeged, Szeged, H-6720, Hungary; b Interdisciplinary Excellence Center, Department of Physical Chemistry and Materials Science, University of Szeged, Szeged, H-6720, Hungary; c HerbaPharm Europe Ltd., Battonya, H-5830, Hungary

**Keywords:** Natural product chemistry, Physical chemistry, Food science, Herbal extracts, Antioxidant activity, Plant processing, DPPH assay, Radical scavenge

## Abstract

Radical scavenging activity of extracts obtained from 16 plants harvested in South Hungary was assessed and compared to the activity of ascorbic acid standard. During extraction, a novel technique involving an ethanolic treatment at ambient temperature was used for advanced active component release. Although the procedure is time consuming, it serves as an efficient and harmless route to extract valuable antioxidant compounds from their natural sources. The as-prepared extracts consist of two phases (except *Allium sativum*), a clear solution and a thick suspension containing solid plant parts that separates in about 2 h. The samples were analysed by the antioxidant assay based on the scavenging of 1,1-diphenyl-2-picrylhydrazyl (DPPH) free radicals. For most of the species, the solid phase retained considerable amount of available antioxidant agents, while the solution parts showed significant radical scavenging activity. The main exceptions were *Nigella sativa, Hippophae rhamnoides* and *Linum usitatissimum*, where the solid parts were less active. Overall, the extracts possessed remarkable antioxidant activity that were compared to published literature data and were found to be superior.

## 1 Introduction

One of the main achievements of modern health conscious lifestyle is the growing interest towards medicine, dietary supplements and food additives of natural origin, such as herbal extracts, which contain high amount of antioxidants or vitamins, depending on the source of plant organs (van der Goot et al., 2016). Antioxidants mainly help maintaining the ideal balance of radicals in cells, preventing oxidative stress related illnesses (Lin and Beal, 2006). Certain vitamins can also assist this goal (e.g., ascorbic acid and tocopherols), while serving other purposes in the body (sight, bone growth, metabolism, blood coagulation, biosynthesis of molecules, etc.). The lack or surplus of antioxidants and vitamins may equally lead to health problems that should be avoided, but herbal extracts are common tools to provide the recommended doses of these vital compounds.

In the past, the antioxidant potency of numerous plants, herbs and spices was reported. One of the most effective representatives is common walnut (*Juglans regia*). It was shown that both its green hull (Schott, 2013) and the nut possess significant activity (Fukuda et al., 2003; Liu et al., 2016) and the hull has antibacterial properties as well. The most important compounds responsible for the antioxidant effect are peptides and polyphenols including tannins. Besides, the leaves of maidenhair tree (*Ginkgo biloba*) are also excellent sources of antioxidants, although the extract from the tree is more known for its remedial effects in treatment of dementia (LeBars et al., 1997).

The seeds of medicinal herbs are also concentrated sources of beneficial components. These plants include milk thistle (*Silybum marianum* (Wojdylo et al., 2007)), mustard (*Brassica juncea* (Katsube et al., 2004)), anise (*Pimpinella anisum* (Hinneburg et al., 2006)), guava (*Psidium guajava*, with flesh (Lim et al., 2007)), caraway (*Carum carvi*), coriander (*Coriandrum sativum* (Zheng and Wang, 2001)), etc. Edible fruits also contain large amount of antioxidants in combination with vitamins (mainly vitamin C) and polyphenols. Some of the most active ones are papaya (*Carica papaya*), guava (*Psidium guajava* (Lim et al., 2007)), sea-buckthorn (*Hippophae rhamnoides* (Wei et al., 2019)), fig (*Ficus carica*), persimmon (*Diospyros kaki* (Katsube et al., 2004)) and various berries (Hakkinen et al., 1999).

Moreover, fragrant herbs are often valuable resources of antioxidant compounds. They can be divided into subgroups such as culinary green herbs, e.g., rosemary (*Rosmarinus officinalis* (Visentin et al., 2012)), lemon balm (*Melissa officinalis* (Wojdylo et al., 2007)), parsley (*Petroselinum crispum* (Hinneburg et al., 2006)), mints (*Mentha* (Zheng and Wang, 2001)), roots, e.g., turmeric (*Curcuma longa*) and ginger (*Zingiber officinale* (Katsube et al., 2004)) and the onion genus, e.g., Chinese leek (*Allium tuberosum* (Katsube et al., 2004)) and garlic (*Allium sativum* (Amagase et al., 2001)). The members of the last group are unique, as they contain notable amount of sulphur compounds that are responsible for their joint antioxidant and antimicrobial character. Furthermore, the antioxidant property of chocolates with high cocoa content was also demonstrated (Medeiros et al., 2015).

In our contribution, 16 plants (Table 1), grown in South Hungary were harvested, dried and treated with a novel type extraction method to achieve high degree extraction of antioxidants of long shelf-life. The obtained extracts were characterized by means of probe reactions to assess their radical scavenging activity.

**Table 1:** The plants and their parts used for the extract preparation and the corresponding EC_50_ (normalized to dried plant mass) and AAEQ values.

Plant	Part used	EC_50_ (μg)/AAEQ (upper phase)	EC_50_ (μg)/AAEQ (mixed)
Common walnut *Juglans regia*	Nut	0.27/38.31	0.24/44.18
Sea-buckthorn *Hippophae rhamnoides*	Seed	0.72/14.46	0.85/12.30
Maidenhair tree *Ginkgo biloba*	Leaf	2.52/4.13	1.46/7.12
Black caraway *Nigella sativa*	Seed	1.52/6.83	1.55/6.72
Horse-chestnut *Aesculus hippocastanum*	Nut	4.94/2.10	2.26/4.60
Milk thistle *Silybum marianum*	Seed	3.77/2.76	2.84/3.66
Common marigold *Calendula officinalis*	Petal	3.78/2.75	2.89/3.60
Ginger *Zingiber officinale*	Rhizome	4.62/2.25	3.91/2.66
Hemp *Cannabis sativa*	Seed	5.19/2.00	4.51/2.31
Caraway *Carum carvi*	Seed	5.85/1.78	4.86/2.14
Sweet wormwood *Artemisia annua*	Leaf	6.60/1.58	5.13/2.03
Linseed *Linum usitatissimum*	Seed	5.46/1.90	7.40/1.40
Bitter melon *Momordica charantia*	Seed	17.13/0.61	10.06/1.03
Garlic *Allium sativum*	Bulb	23.69/0.44	23.69/0.44
Soybean *Glycine max*	Bean	–	–
Summer squash *Cucurbita pepo*	Seed	–	–

## 2 Experimental

### 2.1 Herbal extract preparation

The herbal extracts were prepared from 1000 g of dried plant parts milled to 50 μm grain size. The dried materials were hydrated with 200 mL of filtered water and these slurries were completed to 4000 mL with 96% ethanol solution. After 1 month soaking and extraction period, the larger insoluble parts were separated by centrifugation and the samples were portioned to 100 mL glass vials after vigorous mixing.

### 2.2 DPPH activity tests

The antioxidant activity of the extracts was evaluated by the DPPH (1,1-diphenyl-2-picrylhydrazyl) assay (Brand-Williams et al., 1995). Given the fact that the majority of natural antioxidants possess reactive hydrogen atoms, which serve as the reductants, the DPPH assay is a good measure of the standard antioxidant profile. In a typical experiment, 3500 μL of 60 μM methanolic DPPH solution was mixed with 100 μL herbal extract of various concentrations. The transformation between the oxidized (initial, violet) and reduced (end-product, yellow) form of DPPH was followed by recording the absorbance decrease at 517 nm with a Thermo Scientific Genesys S10 spectrophotometer using 10 mm polystyrene cuvettes. The final absorbance values at steady-state were recorded. The remainder of DPPH is the ratio of final (A) and initial absorbance (A_0_) (DPPH% = A/A_0_). The effective concentration (EC_50_), i.e., the mass of the herbal extract needed to decompose 50% of the initial DPPH, was calculated using the DPPH% versus antioxidant concentration curves. For reference, ascorbic acid (AA) was used and the ascorbic acid equivalent (AAEQ) data were calculated from the obtained EC_50_ numbers (AAEQ = EC_50,AA_/EC_50,plant_). The EC_50,AA_ was determined to be 10.4 μg. All chemicals were from VWR International and used in analytical purity. The accuracy of the method is 5%.

## 3 Results and discussion

The mixed extracts possessed a dark colour and the liquor separated into two fractions in 120 min, excepting garlic extract, which appeared as a homogeneous solution. The upper fraction is a clear ethanolic solution, while the lower phase contains small, aggregated plant parts. Both the upper and lower phases were analysed in the radical scavenging reactions.

The plant extracts required different time frames to achieve chemical equilibrium in the DPPH test, these periods were between 10 and 60 min. Since the reactivity of the antioxidant molecules is not uniform, this is a predictable characteristic. First, the upper phases, i.e., clear solutions, were investigated. After the equilibrium was reached at several doses of the extracts, the final DPPH content was expressed in mole percent and plotted against the mass of dried plant (i.e., the theoretical mass of dried plant required to make the volume of extract used for a measurement) in the cuvette. This mass ranged from 0 to 25 μg and was calculated from the extraction process, i.e., the volume of the extract pipetted for a measurement point and the density of the extracts, proven to be about 1 g/mL.

**Fig. 1:**
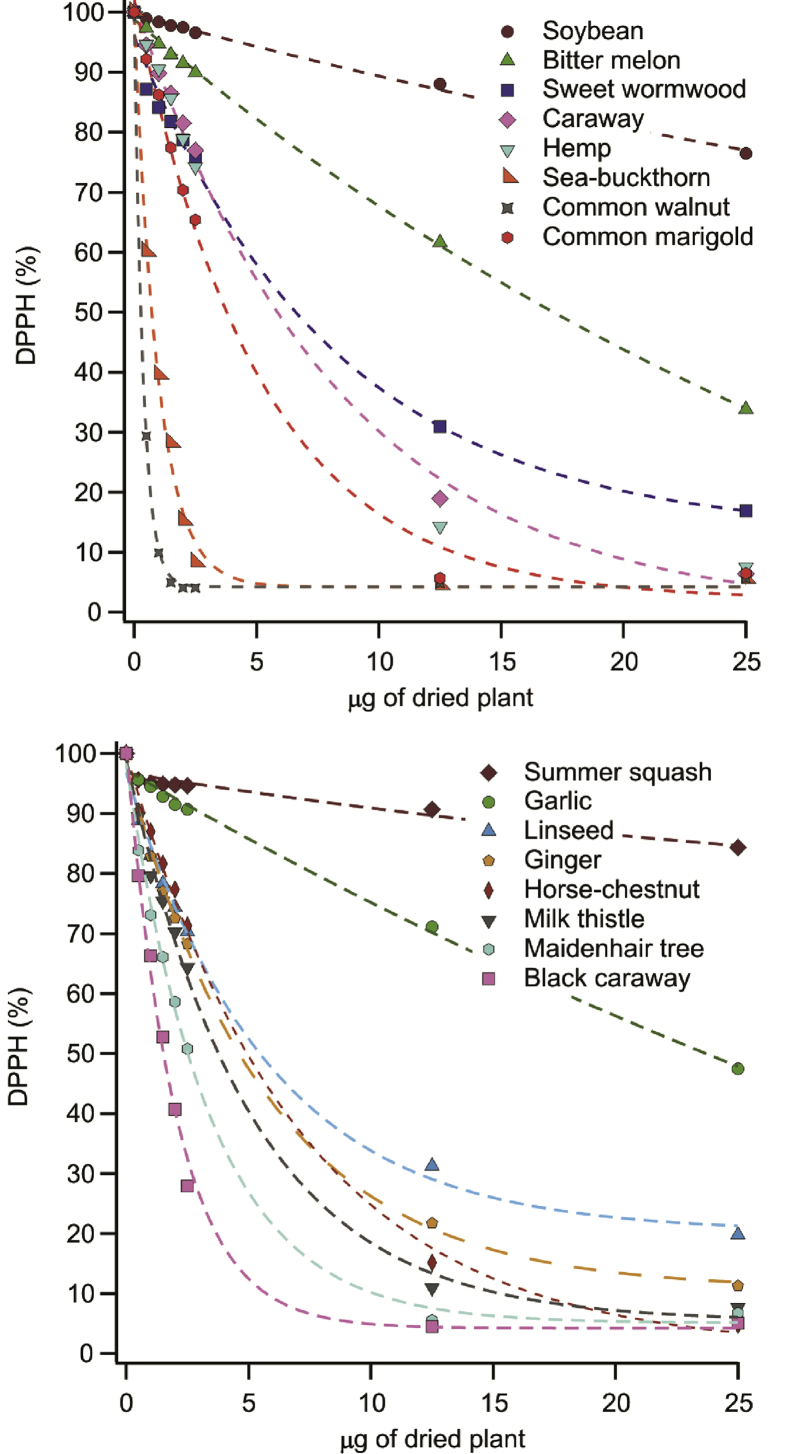
Antioxidant activity expressed by the decrease of DPPH content as a function of the dried plant mass in the solution (upper) phases of the herb extracts investigated.

The DPPH scavenging activity of the plants varied and very poor, poor, mediocre and good scavengers were found, once comparing them together. The decreases of the DPPH concentration for all samples are shown in Fig. 1.

Overall, common walnut was the most effective antioxidant with 0.27 μg EC_50_ value, followed by sea-buckthorn (0.72 μg). The activity of summer squash and soybean was insufficient to calculate the EC_50_ values.

**Fig. 2:**
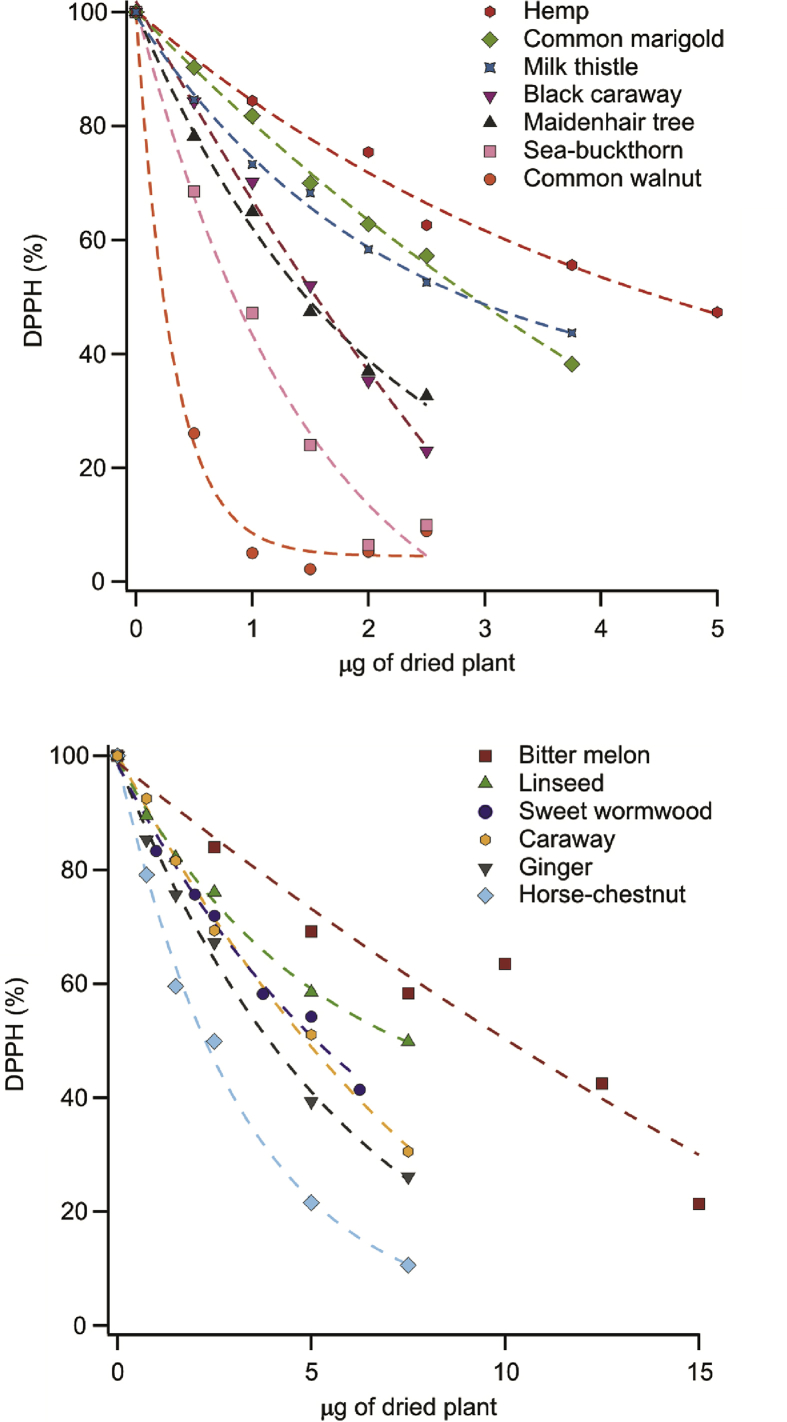
Antioxidant activity expressed by the decrease of DPPH content as a function of the dried plant mass in the mixed herb extracts. Note that garlic consisted of only one clear solution phase, thus mixed extract could not be measured and the mixed extract of soybean and summer squash showed technical difficulties (strong colour) to measure.

**Fig. 3:**
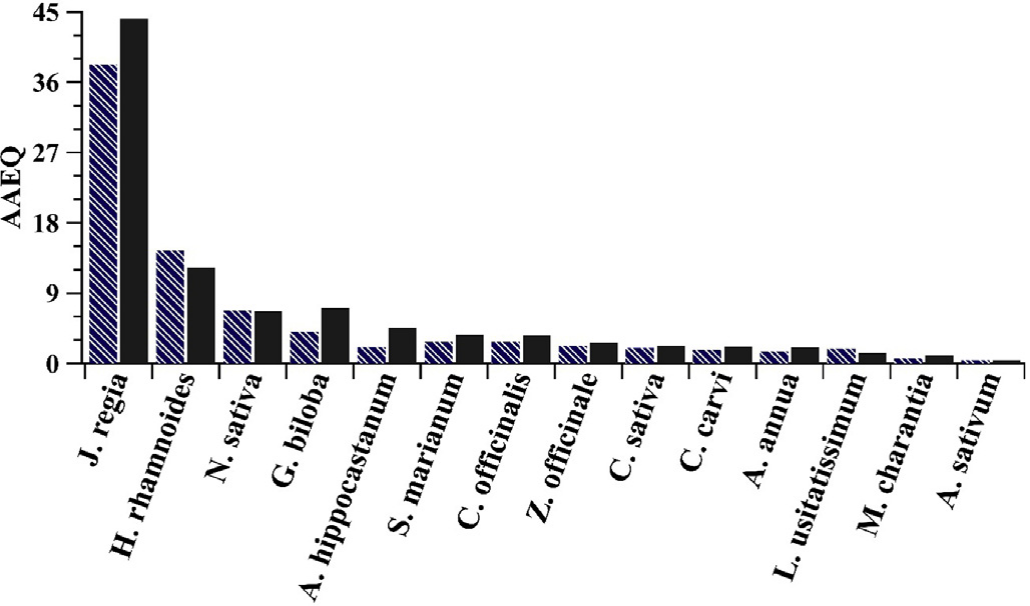
Calculated AAEQ values of the extracts investigated (striped bars belong to the upper phase and full bars refer to the activity of mixed extract).

The experiments were repeated using shaken extracts to access information on the antioxidant activity of the slurry, i.e., the bottom phase, which sediments in long term. If the activity of the two phases combined is higher than the clear supernatants’ one, the undissolved plant parts contain considerable amount of antioxidants that require longer time to express their effect. In the other case, the quantity of remaining antioxidants in the plant parts is low or insignificant. The activity curves of these mixed samples are shown in Fig. 2, while the EC_50_ values together with the AAEQ data for all plants studied are tabulated in Table 1. In addition, the AAEQ values are represented by scale bars in Fig. 3.

The main antioxidant composition of the plants investigated in the present study has been reported before and is detailed in Table 2. The more active plants are known for their widespread antioxidant content. On the other hand, the less active ones may worth a deeper look.

**Table 2:** Antioxidant compounds responsible for the radical scavenging activity of the plants investigated.

Plant	Main antioxidant components (Reference
Common walnut *Juglans regia*	Polyphenols, peptides (Anderson et al., 2001; Fukuda et al., 2003; Liu et al., 2016)
Sea-buckthorn *Hippophae rhamnoides*	Unsaturated fatty acids (Dubey et al., 2018)
Maidenhair tree *Ginkgo biloba*	Flavonoids, glycosides (van Beek, 2002)
Black caraway *Nigella sativa*	Terpenoids, tocopherols (Burits and Bucar, 2000; Trela and Szymanska, 2019)
Horse-chestnut *Aesculus hippocastanum*	Polyphenols (Margina et al., 2015)
Milk thistle *Silybum marianum*	Fatty acids, phenols (Mhamdi et al., 2016)
Common marigold *Calendula officinalis*	Terpenoids (Hamburger et al., 2003)
Ginger *Zingiber officinale*	Phenols (Jolad et al., 2004)
Hemp *Cannabis sativa*	Fatty acids, tocopherols (Oomah et al., 2002)
Caraway *Carum carvi*	Fatty acids, phenols (Ramadan et al., 2003)
Sweet wormwood *Artemisia annua*	Terpenoids (Cavar et al., 2012)
Linseed *Linum usitatissimum*	Tocopherols, polysaccharides (Fedeniuk and Biliaderis, 1994; Trela and Szymanska, 2019)
Bitter melon *Momordica charantia*	Dihydrocarveol (Braca et al., 2008)
Garlic *Allium sativum*	Sulphur compounds, phenols (Lawson and Gardner, 2005; Mnayer et al., 2014)
Soybean *Glycine max*	Isoflavones (Wang and Murphy, 1994)
Summer squash *Cucurbita pepo*	Tocopherols, fatty acids, phenols (Pericin et al., 2009; Rabrenovic et al., 2014)

The bitter melon seeds contain various terpenes, possibly with low antioxidant, but good antimicrobial effect (Braca et al., 2008). The pungent sulphur compounds in garlic are also better known for their antimicrobial activity (Lawson and Gardner, 2005), but their DPPH scavenging ability has also been demonstrated earlier (Mnayer et al., 2014). Soybean, a sample with relatively low antioxidant activity, as indicated by its high EC_50_ value, contains isoflavones, but only up to 5 mg in 1 g of bean (Wang and Murphy, 1994). Furthermore, flavones often react slowly with DPPH radicals, therefore, slow, but longer term activity is foreseen. On the other hand, the highly active extracts such as from common walnuts can be recommended as antioxidant dietary supplement to reduce oxidative stress.

## 4 Conclusions

In the present research, an effective and antioxidant preserving method was developed to obtain highly active antioxidant extracts as ethanolic solutions from plants. The products separated into two phases over time, the radical scavenging activity of both phases was measured. Overall, the plant extracts exhibit remarkable antioxidant properties, from 0.44 (*Allium sativum*) to 44.18 (*Juglans regia*) AAEQ values. Based on their outstanding DPPH scavenging effect and other, already proven benefits, the extracts are promising candidates as commercial food supplements.

## Declarations

### Author contribution statement

Adél Szerlauth: Performed the experiments; Analyzed and interpreted the data.

Szabolcs Muráth: Conceived and designed the experiments; Analyzed and interpreted the data.

Sándor Viski: Contributed reagents, materials, analysis tools or data.

István Szilágyi: Conceived and designed the experiments; Wrote the paper.

### Funding statement

This work was supported by the Hungarian Academy of Sciences (Lendület/96130) and the Ministry of Human Capacities of Hungary (20391-3/2018/FEKUSTRAT).

### Competing interest statement

The authors declare no conflict of interest.

### Additional information

No additional information is available for this paper.
